# Fallopian Tube-Derived Tumor Cells Induce Testosterone Secretion from the Ovary, Increasing Epithelial Proliferation and Invasion

**DOI:** 10.3390/cancers13081925

**Published:** 2021-04-16

**Authors:** Jose A. Colina, Katherine E. Zink, Kanella Eliadis, Reza Salehi, Emma S. Gargus, Sarah R. Wagner, Kristine J. Moss, Seth Baligod, Kailiang Li, Brenna J. Kirkpatrick, Teresa K. Woodruff, Benjamin K. Tsang, Laura M. Sanchez, Joanna E. Burdette

**Affiliations:** 1 Department of Pharmaceutical Sciences, University of Illinois at Chicago, Chicago, IL 60607, USA; jcolin3@uic.edu (J.A.C.); katherineezink@gmail.com (K.E.Z.); keliad2@uic.edu (K.E.); sbalig2@uic.edu (S.B.); li111@uic.edu (K.L.); brennak@uic.edu (B.J.K.); 2 Department of Cellular & Molecular Medicine and Obstetrics & Gynecology, University of Ottawa, Ottawa, ON K1H 8M5, Canada; rsalehi1@ualberta.ca (R.S.); btsang@ohri.ca (B.K.T.); 3 Chronic Disease Program, Ottawa Hospital Research Institute, Ottawa, ON K1Y 4E9, Canada; 4 Department of Obstetrics and Gynecology, Feinberg School of Medicine, Northwestern University, Chicago, IL 60607, USA; emma.gargus@northwestern.edu (E.S.G.); sarah.wagner@northwestern.edu (S.R.W.); kristine.moss@northwestern.edu (K.J.M.); tkw@msu.edu (T.K.W.); 5 Department of Chemistry and Biochemistry, University of California at Santa Cruz, Santa Cruz, CA 95064, USA; lmsanche@ucsc.edu

**Keywords:** fallopian tube, androgen, metabolomics, ovarian cancer

## Abstract

**Simple Summary:**

The area between the fallopian tube and the ovary is of interest since the ends of the fimbria are the progenitor site for high grade serous cancer. Metabolomics revealed that androgens are induced at this site, which then increased proliferation of normal fallopian tube cells and their migration.

**Abstract:**

The fallopian tube epithelium is the site of origin for a majority of high grade serous ovarian carcinomas (HGSOC). The chemical communication between the fallopian tube and the ovary in the development of HGSOC from the fallopian tube is of interest since the fimbriated ends in proximity of the ovary harbor serous tubal intraepithelial carcinoma (STICs). Epidemiological data indicates that androgens play a role in ovarian carcinogenesis; however, the oncogenic impact of androgen exposure on the fallopian tube, or tubal neoplastic precursor lesions, has yet to be explored. In this report, imaging mass spectrometry identified that testosterone is produced by the ovary when exposed to tumorigenic fallopian tube derived PTEN deficient cells. Androgen exposure increased cellular viability, proliferation, and invasion of murine cell models of healthy fallopian tube epithelium and PAX2 deficient models of the preneoplastic secretory cell outgrowths (SCOUTs). Proliferation and invasion induced by androgen was reversed by co-treatment with androgen receptor (AR) antagonist, bicalutamide. Furthermore, ablation of phosphorylated ERK reversed proliferation, but not invasion. Investigation of two hyperandrogenic rodent models of polycystic ovarian syndrome revealed that peripheral administration of androgens does not induce fallopian proliferation in vivo. These data suggest that tumorigenic lesions in the fallopian tube may induce an androgenic microenvironment proximal to the ovary, which may in turn promote proliferation of the fallopian tube epithelium and preneoplastic lesions.

## 1. Introduction

This year an estimated 21,750 women will be diagnosed with ovarian cancer in the United States and an estimated 13,940 will die of the disease [1]. Ninety percent of all ovarian cancer cases are epithelial with high grade serous ovarian cancer (HGSOC) being the most common and deadly subtype of the disease [2,3]. The fallopian tube epithelium (FTE) has emerged as the leading site of origin of HGSOC, thus close scrutiny has been given to the precursor lesions it harbors which may ultimately provide information on early steps in tumorigenesis. Serous tubal intraepithelial carcinomas (STICs) are premalignant lesions formed by neoplastic transformation of the fallopian tube secretory epithelium and are thought to progress into HGSOC. Studies have found that up to 59% of sporadic HGSOC cases had associate STIC lesions [3]. Phosphatase and tensin homolog (PTEN) is absent in 33% of STICs [4,5]. Our lab previously demonstrated that reduction of PTEN expression in murine secretory oviductal cells induced high grade, metastatic tumors that homed to the ovary and the peritoneum in allograft mice and PTEN deleted transgenic mice develop serous tumors in vivo [6,7]. Paired box 2 (PAX2) is a transcription factor that is generally lost in HGSOC and lost in secretory cell outgrowths (SCOUT), a putative precursor lesion in the FTE [8,9,10]. We developed a cell model of a SCOUT via a CRISPR knockout of PAX2 in murine oviductal cells and demonstrated that this model recapitulates salient transcriptional features of human SCOUTs. We further showed that these PAX2 deficient cells are more sensitive to hormone action in vitro [11]. In the present study, we deploy both PTEN deficient and PAX2 deficient oviductal cell lines to study androgen action in fallopian tube.

The role of androgens in the development and progression of epithelial ovarian cancer is documented in epidemiologic and biological studies. The androgen receptor (AR) is expressed in 43–90% of epithelial ovarian cancers with serous cancers being more likely to be AR positive when compared to non-serous [12,13,14,15]. An early study by Helzelsouer et al. showed that elevated levels of the androgens, androstenedione, and dehydroepiandrosterone (DHEA) in patient serum correlated with a higher incidence of ovarian cancer [16]. Women taking testosterone supplements are at an increased risk for developing ovarian cancer [17,18,19]. Anovulation afflicts 95% of women with polycystic ovarian syndrome (PCOS), a prevalent endocrinopathy marked by elevated circulating testosterone [20]. Based on the incessant ovulation hypothesis, one would expect a reduced risk of ovarian cancer in PCOS; however, studies have shown that this patient population is at similar or increased risk of getting ovarian cancer [17,21]. Animal studies have also drawn a connection between androgens and ovarian cancer risk demonstrating that increased androgens can induce proliferation and neoplasia in the ovarian surface epithelium [22] and increase tumor burden in vivo [23].

Androgens have been reported as proliferative [24,25] and pro-migratory in the ovarian surface epithelium and in ovarian cancer cell lines [12,26,27]. Ligr et al. outlined a co-activator role for the phosphorylation of ERK in androgen-induced proliferation and invasion of ovarian surface epithelial and ovarian cancer cells [26]. This AR/MAPK crosstalk is conserved across multiple cell lines including in prostate cancer [28,29]. Despite studies showing the proliferative effects of androgen in ovarian cancer, there is a paucity of data studying androgen action in the fallopian tube epithelium [30]. Limited studies support that the FTE does express AR [26] and that it is responsive to androgen stimulation [31]. We have previously demonstrated that elevated testosterone can have negative reproductive implications on the FTE by impairing cilia beat frequency and repressing the transcription of genes that encode for cilia production. However, given that the site of high grade serous cancer is the secretory epithelium, and that cancer is typically associated with proliferation, this study focused on androgen action in cell lines, oviducts, and human fallopian tube tissues.

Newly developed methods for observing small molecule ovarian secretions using imagining mass spectrometry (IMS) have allowed us to explore novel salpingeal-ovarian metabolomics [32]. In the present study, we demonstrate how this small molecule interaction can shift toward local androgen production in the presence of tumorigenic cell lines derived from the fallopian tube and how this hyperandrogenic microenvironment impacts the FTE. While hyperandrogenism in vivo has mostly been limited to well-established rodent models of PCOS, these models have largely ignored the oviducts and have rarely reported on the androgenic activity in this tissue.

## 2. Materials and Methods

### 2.1. Cell Culture

Murine oviductal epithelial cells (MOE) were isolated and obtained as described previously [33]. MOE cells were maintained in complete medium containing α-MEM modified Eagle’s medium (#10-022-CV, Corning, New York, NY, USA) supplemented with 10% *v*/*v* fetal bovine serum (#100-106, Gemini, West Sacramento, CA, USA), 2 mM l-glutamine (#25030081, Gibco, Waltham, MA, USA), 2 μg/mL epithelial growth factor (#AF-100-15, Peprotech, Cranbury, NJ, USA), 1 mg/mL gentamycin (#30-005-CR, Corning, Corning, NY, USA), 50 U penicillin, 50 μg/mL streptomycin, and 18.2 ng/mL β-estradiol. Stable cell lines were generated by transfection with Lipofectamine 2000 followed by selection with 0.14 µg/mL puromycin (for PTEN small hairpin RNA (shRNA) MOE:PTEN^shRNA^, or scrambled shRNA MOE:SCR^shRNA^). MOE:SCR^shRNA^ and MOE:PTEN^shRNA^ were maintained in similar media but with selection antibiotic. Murine ovarian surface epithelial (MOSE) cells were maintained in media similar to MOE cells except estradiol was not added. For experiments investigating estrogen cells were cultured at least 48 h in “stripped media” consisting of phenol red free α-modified Eagle’s medium (Life Technologies, Carlsbad, CA, USA) supplemented with everything listed above without the β-estradiol and with charcoal stripped FBS.

### 2.2. Mouse Colony and Ovary Removal for Imaging Mass Spectrometry

CD-1 mice were obtained from in-house breeding. Animals were housed in a temperature and light (12L:12D) controlled environment. Water and food were provided ad libitum. All animals were treated in accordance with the National Institutes of Health Guide for the Care and use of Laboratory Animals. Day 16–18 after birth ovaries were removed, dissected free of the uterus, fallopian tube, and bursa using a dissecting microscope (Leica MZ6, Buffalo Grove, IL, USA).

### 2.3. Imaging Mass Spectrometry

Cells and ovary plating, matrix application, and IMS analysis, and statistical analysis using SCiLS (version 2015b, Bruker Daltonics, Billerica, MA, USA) were conducted as previously described [32,34]. An Autoflex Speed LRF MALDI-TOF instrument (Bruker Daltonics) was used to acquire IMS data (using flexControl v 3.4, Bruker) set to the following parameters: Positive reflectron mode, mass range: *m*/*z* 50–2000 Da, raster width: 50 µm. For statistical significance, the Pearson correlation coefficient was set to *p* < 0.05, and no denoising was performed.

### 2.4. Testosterone Extraction

Agarose plugs (300 µL) of the MOE:PTEN^shRNA^/ovarian co-culture condition were collected into 1 mL of 50:50 ACN:H_2_O + 0.1% TFA after four days of incubation at 37 °C and 5% CO_2_. Agarose (including cells and ovarian tissue) and solvent were sonicated for 60 min. Following sonication, the solvent was transferred to a microcentrifuge tube and spun at 10,000 rpm for 10 min to pellet the remaining agarose debris. The crude extract was the further partitioned against CHCl_3_ (3 × 300 µL) to generate a nonpolar (hydrophobic) extract. The resulting nonpolar extract was dried in vacuo.

### 2.5. MS/MS Analysis

MS/MS analysis was performed on an Elute UHPLC interfaced with a Compact Q-TOF (Bruker Daltonics). The nonpolar extract was prepared in MeOH + 0.1% FA (Formic acid) at 10 μg/mL, and a commercial standard of testosterone (T1500, Sigma-Aldrich, St. Louis, MO, USA) was prepared in MeOH + 0.1% FA to 10 mM. Samples (5 µL) were analyzed on a C18 column (C18 Poroshell UPLC 1.9 µm (2.1 × 1.5 mm)) using an isocratic gradient at 60% B (A: H20 + 0.1% FA, B: MeOH + 0.1% FA) over 10 min. The instrument was internally calibrated using ESI Tune Mix (Bruker Daltonics) and set to the following parameters: Mass range: *m*/*z* 50–2000, Positive mode, Auto MS/MS with 9 precursor ions per cycle. Testosterone was fragmented at 35 eV.

### 2.6. Cell Proliferation and Viability

Cells were seeded in 96-well clear, flat-bottom plates (Microtest 96, Falcon, Corning, NY, USA) at a density of 500 cells per well. Each plate was incubated overnight at 37 °C under 5% CO_2_. R1881 and bicalutamide were dissolved in DMSO and diluted to the appropriate concentrations to a total volume of 100 μL and 0.1% DMSO per well. The cells were then incubated for at 37 °C. Cell viability was assessed by Celltiter-Blue assay kit (Promega, Madison, WI, USA) as instructed by the manufacturer. After treatment, cells were incubated with CellTiter-Blue^®^ reagent for 1 h at 37 °C. Fluorescence was measured on a BioTek Synergy 2 microplate reader (BioTek, Winooski, VT, USA) at 560/590 nM. For sulforhodamine B (SRB) assays, MOE cells were plated at 5 × 10^3^ cells/mL in a 96-well plate then treated and incubated for 5 days followed by colorimetric assay as described previously [35]. Absorbance at 505 nM was measured on a BioTek Synergy 2 microplate reader.

### 2.7. Western Blot

Protein lysate (35 μg) was run on SDS-PAGE and transferred to nitrocellulose membrane. Blots were blocked with 5% milk or BSA in TBS-T and probed at 4 °C overnight with primary antibodies (Table S1). Anti-rabbit HRP-linked secondary antibody (Cell Signaling #7074S) was used for 30 min in blocking buffer. Membranes were incubated and developed as described previously [36].

### 2.8. RNA Isolation, cDNA Synthesis and RT-PCR

RNA extraction was performed using Trizol (Life Technologies, Grand Island, NY, USA) and chloroform with isopropanol precipitation followed by ethanol washes and DNAse step. iScript™ cDNA synthesis kit (#170-8841, BioRad, Hercules, CA, USA) and SYBR green (#AB1323A, Thermo Fisher, Waltham, MA, USA) were used according to manufacturer’s instructions. All qRT-PCR measurements were performed using the CFX connect Real-Time PCR Detection System (Bio-Rad). Samples were normalized to the housekeeping gene glyceraldehyde-3-phosphate dehydrogenase (GAPDH). Primers designed using NCBI Primer-Blast tool (https://www.ncbi.nlm.nih.gov/tools/primer-blast/, accessed on 15 March 2021) (Table S2).

### 2.9. Invasion Assay

Matrigel (356234, Corning) was diluted to 300 μg/mL in αMEM media (10-022-CV, Gibco). Then 120 μL of diluted Matrigel was added to each Boyden chamber insert with 8 μm pores (PI8P01250, Millipore, Burlington, MA, USA) and incubated for 1 h at 37 °C. Each cell line was collected with trypsin, counted, centrifuged, and resuspended in αMEM. Each cell line (50,000 cells per insert) was added to the top of each insert. αMEM (500 μL) with either 0.1% DMSO (vehicle control), R1881, bicalutamide, U0126, or combination in DMSO was placed under each Boyden chamber and used as the chemoattractant. After 24 h, the Matrigel and noninvaded cells remaining on top of the inset were removed with a cotton swab. Cells that invaded through the Matrigel and were on the bottom of the inset were fixed with paraformaldehyde, permeabilized with 70% methanol, and stained with crystal violet. Images of stained cells were captured with an inverted microscope and counted with ImageJ.

### 2.10. Dihydrotestosterone (DHT) Treated Rat Model of PCOS

Female Sprague Dawley rats (Charles River, Montreal, QC, Canada) were maintained on 12 h cycle (light and dark) and given food and water ad libitum. All animal procedures were carried out in accordance with the Guidelines for the Care and Use of Laboratory Animals, Canadian Council on Animal Care, and were approved by the University of Ottawa Animal Care Committee. Immature female rats at 21 days of age were implanted subcutaneously with silicone capsules without (control, sham control) or with dihydrotestosterone (DHT, Steraloids Inc., Newport, RI, USA), as previously described [37,38] to continuously release 83 μg DHT/day for 28 days. Sham control animals received identical pellets lacking the steroid. Oviducts from both control and DHT-treated rats were isolated and fixe in paraformaldehyde until analysis.

### 2.11. Dehydroepiandrosterone (DHEA) Treated Mouse Model of PCOS

Female CD-1 mice were housed at Northwestern University’s Center for Comparative Medicine in a temperature, humidity, and light controlled (12 h light/12 h dark) environment. Animals were fed irradiated 2919 chow, which does not contain soybean or alfalfa meal to minimize the exposure to phytoestrogens. Five mice were housed per cage and food and water were provided ad libitum. All animal experiments were approved by the Institutional Animal Care and Use Committee and were performed in accordance with the National Institutes of Health Guidelines and public law. Female pre-pubertal (post-natal day 25) mice were randomly divided into two groups (control group and DHEA group; *n* = 10 per group for each of two replicates). A stock solution (300 mg/mL) of DHEA was prepared in 100% histology-grade ethanol, which was then diluted in sesame oil to make a working solution (1:20 dilution, final concentration 15 mg/mL). Animals of the DHEA group were injected daily with 60 mg/kg body weight DHEA in sesame oil (100–200 µL) subcutaneously for 20 consecutive days [39]. The control group animals were administered 1:20 ethanol in sesame oil (100–200 µL) for 20 consecutive days. The location of the injection site was varied to avoid leakage or irritation. Throughout the treatment period, animals were weighed daily. Beginning on the 10th day of injections, vaginal lavage was performed daily. Mice were sacrificed on the afternoon of the 20th day of injections. Blood was collected by cardiac puncture under anesthesia. Immediately following blood collection, mice were euthanized by cervical dislocation. Oviductal tissue from one side were fixed in modified Davidson’s fixative overnight at 4 °C.

### 2.12. Statistical Analysis

Data are presented as mean ± standard error, with significance determined utilizing GraphPad Prism software (GraphPad, La Jolla, CA, USA) for *p* ≤ 0.05. All data sets were analyzed for significant outliers by Grubbs’ test of deviation. All conditions were tested in three replicates in at least triplicate experiments. Statistical significance was determined by Student’s *t*-test, one-way ANOVA, or two-way ANOVA with Tukey’s or Dunnett’s post-hoc test. Specific statistical method used for each figure is specified in figure legend. * *p* < 0.05 was considered statistically significant.

## 3. Results

### 3.1. Ovarian Explant/MOE:PTEN^shRNA^ Co-Culture Significantly Upregulates Ovarian Production of Testosterone

**Figure 1:**
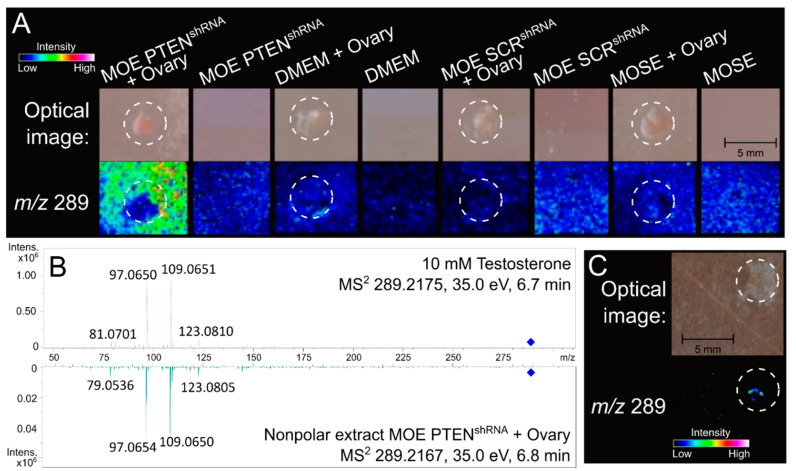
Tumor cells increase ovarian production of androgens. (**A**) A biomolecule upregulated in an in vitro tumorigenic condition may be testosterone. A validated imaging mass spectrometry sample preparation method was employed to detect increased production of small molecules (<2000 Da) in a condition of tumorigenic murine oviductal epithelial cells (MOE PTEN^shRNA^) incubated with a healthy murine ovary, compared to seven controls. Signal detection (by nominal *m*/*z*) was compared to MOE:SCR^shRNA^ (MOE cells with a scrambled shRNA, used as a gene control), MOSE (murine ovarian surface epithelial cells, used as a cell control), and culture medium, DMEM. Each cell line was incubated for four days with and without an ovary (outlined by a dotted white circle). Resulting IMS data were normalized to the TIC and evaluated by the statistical software SCiLS to determine signals significantly more abundant (*p* < 0.05) in the MOE PTEN^shRNA^ + Ovary condition compared to all others. One resulting signal was *m*/*z* 289, which putatively matched the nominal mass of protonated testosterone (exact calculated mass, [M + H]^+^ = *m*/*z* 289.2168). (**B**) Testosterone presence was validated using tandem mass spectrometry. Direct mass fragmentation comparison of a testosterone standard (T4, 10 mM) to an extract of the MOE PTEN^shRNA^ + Ovary sample validated the identity of the *m*/*z* 289 in the IMS panel as testosterone. The standard (2.4 ppm error) and extracted testosterone (0.34 ppm error) shared four matching mass fragments, generating confidence as a Level One identification according to the Chemical Analysis Working Group (CAWG). (**C**) Testosterone is produced by the ovary. A divided chamber setup, previously discussed [32], was employed to determine the source of the testosterone production. Incubation conditions were identical and spatial orientation was tested with the MOE PTEN^shRNA^ cell culture (bottom left half) distanced by ~2 mm from the ovary explant (top right), which was plated in media to maintain growth conditions. The *m*/*z* 289 was distinctly detected around the ovarian tissue, indicated that the ovary produces or releases testosterone when incubated in the presence of tumorigenic MOE cells.

Only the fimbriated ends of the fallopian tube that are in proximity of the ovary display serous tubal intraepithelial carcinoma in situ, a lesion thought to give rise to high grade serous cancer. Endocrine signals from the ovary also play a role in physiological changes in the FTE to support functions such as gamete and embryo transport, tubal secretions, and sperm capacitation [40,41,42,43]. Previously, we have shown that tumorigenic fallopian tube cells can differentially modify the metabolome of the ovary as compared to nontumorigenic controls. Murine oviductal epithelial (MOE) cells derived from secretory cells were co-cultured with murine ovarian explants. MOE:PTEN^shRNA^, MOE:SCR^shRNA^, and murine ovarian surface epithelial cells (MOSE) cells were cultured with and without a murine ovarian explant for 4 days (Figure 1A). MOE:SCR^shRNA^/ovarian co-culture served as a control for any secretions that would normally be induced by the presence of healthy FTE, while the MOSE/ovarian co-culture served as a control for any small molecules that may be enriched simply by cells of non-fallopian tube origin. Imaging mass spectrometry was conducted, and mass error was calculated for each run. Identified signals were adjusted post acquisition to incorporate the mass shifts based on matrix peaks as previously described [32]. Signals that were uniquely enriched in the MOE:PTEN^shRNA^/ovarian co-culture condition compared to the seven control conditions were prioritized for identification using the Human Metabolome Database using a nominal mass search [44]. One signal at *m*/*z* 289 matched protonated testosterone ([M + H]+ = *m*/*z* 289.2168). Further analysis of the co-culture extract was conducted via tandem MS/MS (Figure 1B). A commercial testosterone positive control (10 mM) and the nonpolar co-culture extract shared four diagnostic ESI fragments and matched in retention time, confirming presence of the androgen in the extract. A direct comparison of a standard to an extract with error the precursor (intact) ions below 5 ppm lends confidence to assign a “Level One” identification of the molecule as testosterone, according to the guidelines of the Chemical Analysis Working Group (CAWG) Metabolomics Standards Initiative [45]. Given the experimental set up of the original IMS experiment, the data generated by the co-culture layout were only useful in determining the presence or absence of a particular signal in a condition, not in illuminating the source of the biomolecular production or release. To confirm the origin of the testosterone rich MOE:PTEN^shRNA^/ovarian co-culture extract, a divided chamber experiment that segregated the cells from the ovarian explant was developed and incubated in the same manner as the spatially independent co-culture conditions (Figure 1C). The spatial resolution afforded by IMS revealed enrichment of testosterone exclusively within the ovarian explant and not surrounding the MOE:PTEN^shRNA^ cells, although production of testosterone was influenced by the proximal presence of the tumorigenic cells.

### 3.2. Androgen Increases In Vitro Proliferation of Murine Oviductal Epithelial Cells

**Figure 2:**
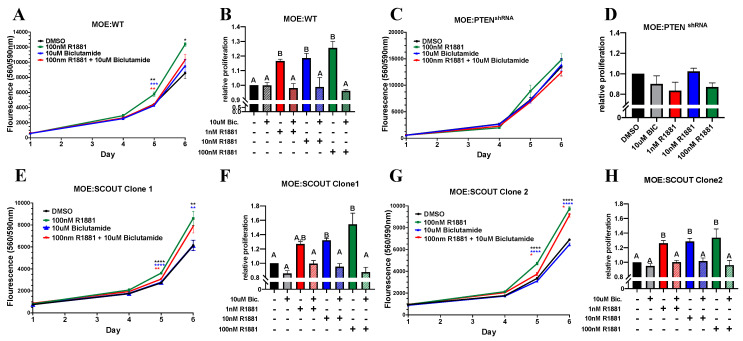
Androgens increase in vitro proliferation of murine oviductal epithelial cells. (**A**,**C**,**E**,**G**) Cell viability was measured by CellTiter Blue assay after exposure to DMSO, 100 nM R1881, 10 μM bicalutamide, or a combination for 4, 5, and 6 days (each day was individually analyzed using a one-way ANOVA followed by a Dunnett’s post hoc, *n* ≥ 4, Significant differences from control are represented by * *p* < 0.05, ** *p* < 0.01, *** *p* < 0.001, **** *p* < 0.0001). (**B**,**D**,**F**,**H**) Cells were cultured in various concentrations of R1881 with and without 10 μM bicalutamide for 5 days. Relative proliferation was measured via Sulforhodamine B (SRB) assay (*n* ≥ 3, one-way ANOVA followed by a Tukey’s post-hoc where *p* < 0.05, letters above bars indicate differences between groups at *p* < 0.05). Data displayed as mean ± SEM.

Precursor lesions to high grade serous ovarian cancer and STICs can be found in close proximity to the ovary in the fimbriated end of the fallopian tube. Given our finding that PTEN deficient epithelial cells, which form tumors in mice, can uniquely induce testosterone secretion from the ovary, we sought to understand how androgens may impact proliferation. Wild-type secretory murine fallopian tube secretory epithelial cells (murine oviductal epithelium, MOE:WT) or two independent clones of a secretory cell outgrowths generated by CRISPR deletion of PAX2 (MOE:SCOUT) [11] were treated with androgens. To isolate androgen receptor mediated changes in proliferation, cells were treated with the synthetic androgen receptor agonist R1881. MOE:WT and both MOE:SCOUT clones showed a significant increase cell proliferation after exposure to R1881 (100 nM) after 5 days compared to vehicle treated control (Figure 2A,E,G). Cell growth was reversed by cotreatment with 10 μM of the selective androgen receptor antagonist, bicalutamide. Furthermore, R1881 proliferation was dose dependent in MOE:WT and MOE:SCOUT cells (Figure 2B,F,H) ( Figure S1A). MOE:SCOUT Clone 1 & 2 showed a higher induction of proliferation (54.5% ± 15.5% & 33.7% ± 11.9% respectively) compared to MOE:WT (25.5% SEM ± 4.5%). While MOE:PTEN^shRNA^ cells increased ovarian secretion of testosterone in the IMS data and express androgen receptor, these cells failed to respond to R1881 based on proliferation at any concentration tested (Figure 2C,D) (Figure S1B,C).

### 3.3. Androgens Mediated Proliferation Is Dependent on Presence of Phosphorylated ERK (pERK)

**Figure 3:**
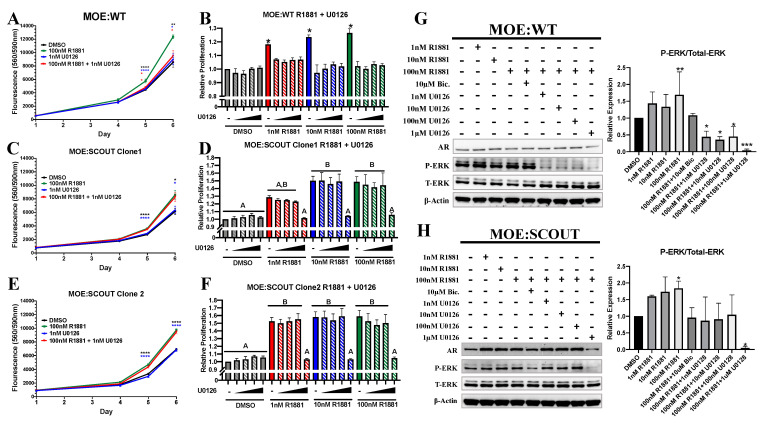
Androgens mediated proliferation is dependent on presence of phospho-ERK. (**A**,**C**,**E**) Cellular viability was measured by CellTiter Blue assay after exposure to DMSO, 100 nM R1881, 1 nM U0126, or a combination for 4, 5, and 6 days (each day was individually analyzed using a one-way ANOVA followed by a Dunnett’s post hoc, *n* ≥ 4, Significant differences from control are represented by * *p* < 0.05, ** *p* < 0.01, *** *p* < 0.001, **** *p* < 0.0001). (**B**,**D**,**F**) Cells were cultured in various concentrations of R1881 (0 nM, 1 nM, 10 nM, and 100 nM, respectively) and U0126 (0 nM, 1 nM, 10 nM, 100 nM, and 1 μM respectively) for 5 days. Relative proliferation was measured via Sulforhodamine B (SRB) assay (*n* ≥ 3, one-way ANOVA followed by a Tukey’s post-hoc, letters above bars indicate differences between groups at *p* < 0.05). (**G**,**H**) Western blots and relative densitometry for MOE:WT and MOE:SCOUT clones cultured in steroid free media for 24 h followed by treatment with various concentrations of R1881 and U0126 for 24 h.

ERK is a putative AR coactivator [46], and AR stimulation has been reported to lead to activation of MAPK signaling [29,47]. Blocking ERK phosphorylation has been shown to reduce AR gene transcription [28], and it has been demonstrated that pERK is necessary for androgen-induced proliferation of ovarian cancer cell lines OVCAR3 and SKOV3 cells [26]. To determine whether phosphorylation of ERK is required for androgen-induced proliferation in MOE cells, we treated cells with the MEK(ERK kinase) inhibitor, U0126, alone or in combination with R1881. Cellular viability of MOE:WT increased with exposure to 100 nM R1881 after days 5 and 6 compared to DMSO control and this was reversed by co-treatment with U0126 (Figure 3A). R1881 induced proliferation after 5 days was also ablated by cotreatment with as little as 1 nM U0126 (Figure 3B). Both MOE:SCOUT clones did not show repression of cellular viability when co-treated with 1 nM U0126 and R1881 (Figure 3C,E). Instead, these models required exposure to 1 μM U0126 to reach significant reduction of R1881 induced proliferation in the SCOUT clones (Figure 3D,F). In MOE:WT cells 1 nM of U0126 reduced phosphorylation of ERK while in MOE:SCOUTs 1 μM of U0126 was required (Figure 3G,H). Lastly, 100 nM R1881 did not increase the ability of MOE cells to form 2D colonies; however, it did increase total colony area. The effect was abolished by co-treatment with U0126 (Figure S1D).

### 3.4. Androgens Increase Invasion and Mesenchymal Markers of Murine Oviductal Epithelial Cells

**Figure 4:**
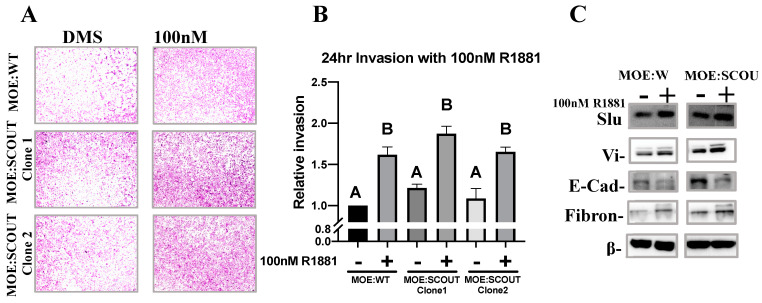
Androgen increases in vitro invasion of murine oviductal epithelial cells. (**A**,**B**) Cellular invasion was measured by Boyden chamber assay. MOE:WT or MOE:SCOUT cells were seeded in steroid free media on Matrigel coated transwells and allowed to invade toward steroid free media supplemented with either DMSO or 100 nM R1881 for 24 h. (*n* ≥ 3, one-way ANOVA followed by a Tukey’s post-hoc, letters above bars indicate differences between groups at *p* < 0.05). (**C**) Western blot analysis of markers associated with invasion. Cells were cultured in steroid free media for 24 h followed by exposure to either DMSO or 100 nM R1881 for 24 h.

The colonization of fallopian epithelial cells from the fimbriae onto the ovary, whether by precursor escape or metastasis of tumorigenic lesions, is thought to be a critical step in the progression of HGSOC. Androgen receptor agonists have been shown to induce migration and invasion in vitro in a variety of cell lines including ovarian cancer cells [26]. Initially, we investigated the impact of androgen stimulation on migration using MOE:WT and MOE:SCOUT cell lines via an in vitro scratch assay. R1881 showed no significant increase in migration compared to DMSO control in either cell lines. Subsequently, we explored the role of androgen stimulation on invasion through a 3-dimensional matrix. To accomplish this MOE:WT and MOE:SCOUT models were tested in a Boyden chamber assay. MOE:WT and MOE:SCOUT cells both showed a significant increase in invasion through Matrigel after just 24 h of exposure to 100 nM R1881 compared to control (Figure 4A,B). Furthermore, after just 24 h of R1881 exposure, MOE:WT and MOE:SCOUTs showed higher expression of mesenchymal markers slug, vimentin, and fibronectin and lower expression of epithelial marker E-cadherin based on western blot analysis (Figure 4C). Previous reports have shown that androgen induced invasion can also be mediated by pERK and increased MMP2 [48]. However, U0126 (1 μM) did not reverse the invasion seen in the Boyden chamber assay nor did R1881 increase MMP2 expression in MOE cells (Figures S2 and S3).

### 3.5. Ablation of pERK Partially Reduces Expression of AR Target Genes

**Figure 5:**
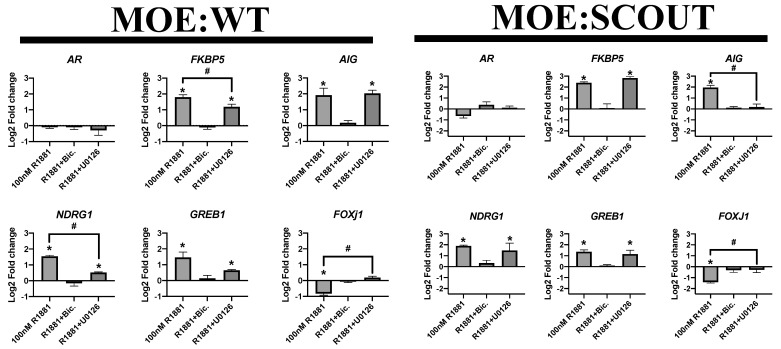
U0126 blocks transcription of some, but not all, putative AR target genes in fallopian tube cells. qPCR analysis of select androgen responsive genes shown as Log_2_ fold change compared to DMSO control. (*n* ≥ 3, one-way ANOVA followed by a Dunnett’s post-hoc; * *p* < 0.05; Additionally, unpaired *t*-test comparing cells treated with 100 nM R1881 to 100 nM R1881 + 1 μM U0126 was performed; # *p* < 0.05).

Previous studies have found that ERK directly interacts with the androgen receptor and enhances AR target gene transcription [46]. Blocking the MAPK signaling pathway can reverse ligand dependent and independent activation of an ARE-inducible reporter gene [28,46]. We hypothesized that phosphorylated ERK mediated genomic activity of the androgen receptor in the fallopian tube. MOE:WT and MOE:SCOUT cells were co-treated with R1881 and U0126 and observed transcriptional activity downstream of androgen receptor by qPCR. Six putative AR target genes were selected (FKBP5, AIG, NDRG1, GREB1) along with FOXJ1. In MOE:WT cells, U0126 (1 μM) was able to completely ablate liganded AR downregulation of FOXJ1 and partially reverse liganded AR upregulation of NDRG1 and FKBP5 (Figure 5). Similarly in MOE:SCOUT cells, U0126 reversed downregulation of FOXJ1 and reversed R1881 induced upregulation of AIG (Figure 5). However, in neither cell line was U0126 able to reverse R1881 induced upregulation of GREB1 and only partially reverse upregulation of FKBP5 and NDRG1.

### 3.6. Rodent Models of PCOS Do Not Exhibit Epithelial Proliferation in the Oviducts

**Figure 6:**
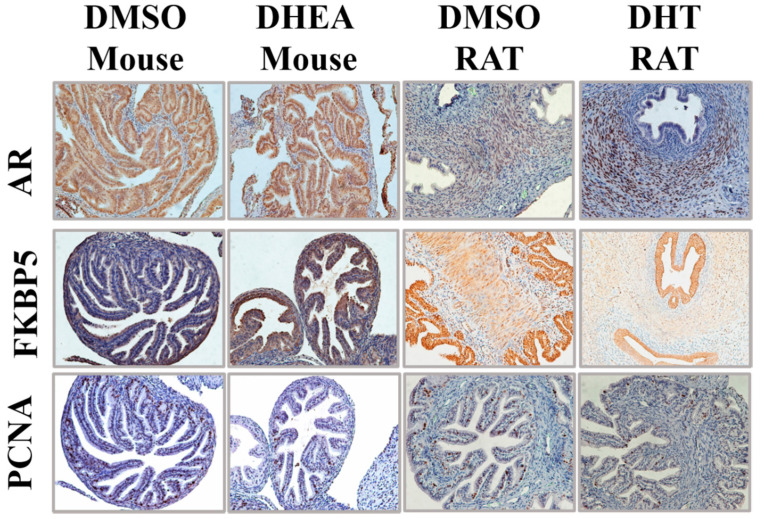
Rodent models of PCOS do not exhibit epithelial proliferation in the oviducts. Oviducts of DMSO or DHEA treated mice and DMSO or DHT treated rats were serially sectioned and stained for antibodies against androgen receptor (AR), FKBP5, or PCNA. Scale bar represents 100 μm.

Given the proliferative effects of androgens on MOE cells observed in vitro, we sought to characterize the effects of hyperandrogenism on the oviduct in vivo. To achieve this, we performed immunohistochemistry on the oviducts of two rodent models that are supplemented with androgen or precursor hormones to generate a PCOS-like phenotype (Figure 6). Androgen supplemented rodents have long been used for PCOS research on the ovary and adipose, however these studies have yet to characterize the androgenic effects on the oviducts.

In the dihydrotestosterone (DHT)-rat model, 21-day old female rodents are given 83 μg of DHT per day for 28 days before being sacrificed. This model has been shown to recapitulate salient metabolic and ovarian features of PCOS seen in patients and exhibit 1.7-fold higher plasma DHT levels compared to control [37]. The DHT rat oviducts had a significant increase in nuclear AR expression only in the stroma, while the epithelium expressed very little AR. Surprisingly, the DHT treated rats had reduced FKPB5 expression in the epithelium along with reduced PCNA staining overall.

In addition to the DHT-rat model, we analyzed the oviducts of Dehydroepiandrosterone (DHEA) treated mice, another commonly used model of PCOS. DHEA is an androgenic intermediate that is converted to testosterone in adrenal and gonadal steroidogenesis, and DHEA is increased in women with PCOS [39,49]. Female mice (25 days old) were injected with 60 mg/kg DHEA for 20 days before being sacrificed. This model has been shown to recapitulate various features of PCOS including, acyclicity, anovulation, polycystic ovaries, and hyperandrogenism [39]. In the DHEA-mouse model we observed slightly higher cytoplasmic and nuclear androgen receptor staining in the epithelium along with higher expression of putative androgen receptor target FKBP5; however, PCNA, a marker of proliferation was reduced in the epithelium of these tissues. These data support an androgenic, but not proliferative activity, in the oviductal epithelium of these DHEA treated mice.

## 4. Discussion

The fimbriated ends of the fallopian tube that often touch and directly interact with the ovary are the site of serous tubal intraepithelial carcinoma that likely gives rise to high grade serous cancer. This is the first report to show that PTEN^shRNA^ engineered fallopian tube derived tumor cells, when grown in proximity of the ovary, can induce ovarian production of testosterone possibly creating a local androgenic microenvironment around the ovary and the fimbria. This study also found that exposure to testosterone was sufficient to increase proliferation and invasion of fallopian tube secretory cell lines and models of secretory cell outgrowths.

We have previously demonstrated that ovarian metabolites can be altered in the presence of fallopian precursor lesions through bidirectional endocrine crosstalk [32]. Normal ovarian steroidogenesis occurs in the theca and granulosa cells where cholesterol is metabolized into progesterone, estradiol, and various androgenic intermediates. Androgen production and secretion changes with the menstrual cycle and has been shown to support maturation of antral follicles. This process is regulated by luteinizing hormone (LH), along with various other growth factors and hormones, that alter expression levels of metabolic enzymes involved in steroidogenesis. Previously, enhanced ovarian production of androgens has been attributed, in large part, to theca overexpression of metabolic enzyme cytochrome P450c17, enhanced signaling through LH receptors, granulosa cell hypersensitivity to FSH, and inflammation caused by biomechanical pressure from stromal hypertrophy and cortical thickening [49]. Here, IMS demonstrated that tumorigenic cells of fallopian tube origin can induce ovarian androgen production as compared to nontumorigenic controls and non-fallopian tube derived cells (Figure 1 and Figure S4). It is not yet known what signals the PTEN deficient oviductal cells release that allow for the change in hormonal ovarian secretion of androgen. Identification of this signal could provide a target to specifically ablate fallopian tube tumor cell induced metabolomic secretions from the ovary. Furthermore, future IMS studies will be needed to elucidate whether benign fallopian precursor lesions, such as SCOUTs or p53 signatures, can also increase androgen secretion from the ovary.

We set out to characterize the oviducts of two commonly used rodent PCOS models in order to provide more context for the pro-proliferative phenotypes observed in vitro. Importantly, while the ovaries and uteri of PCOS-like rodent models have been extensively reported on in the literature, the oviduct has never been characterized. The DHT treated rat model exhibited more nuclear androgen receptor expression in the stroma than in the epithelium. In fact, compared to control, the DHT treated rat had reduced expression of the AR target gene, FKBP5. The lack of epithelial androgen activity in either the treated or untreated rats makes this model less suitable for studying the effects of androgens on the development of fallopian tube derived HGSOC. This model can, however, support research into the effects of PCOS-induced hyperandrogenism on the uterus, which has been reported to show a similar AR staining profile as the oviducts in terms of stromal expression [31]. The DHEA treated mouse showed higher staining of AR downstream target FKBP5 in the oviductal epithelium. Although opposite of our in vitro findings, the epithelium of the treated mice was less proliferative than the control. PCOS may not fully capture the effects of acute local hyperandrogenism experienced by the fimbriated FTE as evidenced by our in vitro data. These data underscore the need for further research into developing models that can accurately recapitulate endocrine cross talk in the reproductive tract without peripheral hormonal administration.

Given what is known about the proliferative action of androgens, we investigated how activation of the androgen receptor may impact proliferation of the fallopian tube epithelium. MOE:WT cells, a murine model of healthy FTE, and MOE:SCOUTs proliferate in response to 100 nM R1881 consistent with previous studies showing that the ovarian surface epithelium proliferates to a similar degree in response to a synthetic androgen [24]. Interestingly, MOE:PTEN^shRNA^ induced testosterone when grown in proximity of the ovary, but were not susceptible to androgen-induced proliferation, nor were they effected by bicalutamide. This data is consistent with existing studies that have examined the interactions between PTEN and AR in breast and prostate cancer showing that PTEN and AR directly interact and that loss of PTEN, and commensurate increased PI3K activity, reduces AR signaling [50,51,52]. While our data suggests that PTEN deficient tubal cells may themselves be insulated from the pro-proliferative effects of ovarian androgenic secretions, the surrounding epithelium and earlier precursor lesions such as SCOUTs remain susceptible. Our data further suggests that androgens can act as a migratory chemoattractant consistent with previous results showing that ovarian cancer cells lines invade through Matrigel better when exposed to androgens.

There is an abundance of research implicating aberrant activation of the MAPK signaling cascade pathway in proliferation, survival, differentiation, and apoptosis of many types of cancer. Further, a suite of inhibitors for this pathway are approved to treat skin, kidney, liver, lung, and colon cancer [53]. ERK1/2, a key propagator of the MAPK kinase signaling cascade, has been shown to be constitutively active in epithelial ovarian cancer and is necessary for hormone induced proliferation and invasion of OVACR-3 and SKOV-3 cells [26]. Our data expands on these previous studies demonstrating that blocking phosphorylation of ERK1/2 was able to repress androgen induced proliferation in the fallopian tube. Despite MOE:WT and MOE:SCOUT exhibiting similar amounts of total- and phospho-ERK1/2 under untreated conditions (Figures S2 and S5), higher concentrations of MEK inhibitor U0126 was needed to block androgen induced proliferation and ablate ERK phosphorylation in MOE:SCOUT cells. Our lab found that PAX2 re-expression in MOE:PTEN^shRNA^ cells results in suppression of p70S6K [54]. Activated p70S6K has been shown to mediate cross talk between mTOR and MAPK signaling and may represent an orthogonal route for preserving ERK phosphorylation in MOE:SCOUT cells, overcoming upstream MEK inhibition [55]. Lastly, qPCR analysis of AR target genes showed that U0126 reversed AR transcriptional activity of some, but not all, of the selected genes (Figure S3). This may explain why, in the cell lines studied here, U0126 was able to reverse androgen induced proliferation but not invasion, because unique subsets of genes are involved in each phenotype.

Androgen deprivation therapy is among the first-line treatment options for aggressive, castration resistant prostate cancer; a recent meta-analysis showed that second-generation, non-steroidal antiandrogens such as enzalutamide significantly improve overall survival and metastasis free survival in these patients [56,57]. Anti-androgen therapy in breast and endometrial cancer has also been explored with Phase II clinical trials of finding that treatment of these compounds in a sub-set of patients resulted in a clinical benefit rate of 19% and 33%, respectively [58,59,60]. Research into the use of anti-androgens in ovarian cancer has seen mixed results. In vitro experiments, including the data presented here, support the effectiveness of anti-androgens as a means for reversing androgen induced proliferation, invasion, and survival in premalignant fallopian tube cells, ovarian surface epithelial cells, and primary ascitic ovarian cancer cells [61,62,63]. One study used an OVCAR3 xenograft mouse model and demonstrated that androgen treatment increased tumor growth and co-treatment with enzalutamide rescued the effect [64]. Despite the success of these pre-clinical data, multiple phase II clinical trials of anti-androgens as a treatment for ovarian cancer have only shown a modest effect. Three Phase II trials have been conducted using first-generation anti-androgens flutamide or bicalutamide; none of these trials reached their predetermined endpoints for clinical significance [65,66,67]. There is currently an ongoing Phase II study evaluating the use of enzalutamide on AR+ ovarian cancer (NCT01974765). Furthermore, the data presented here indicate that healthy FTE and fallopian precursor lesions are susceptible to oncogenic effects of androgens and are responsive to anti-androgens. Gucalp et al. highlight the shortcomings of simply using the criteria of AR+ IHC staining to determine patient inclusion in anti-androgen clinical trials [58].

## 5. Conclusions

The data presented here shows that neoplastic lesions in the fallopian tube can elicit unique ovarian secretions; more research will need to be done to determine if these secretory signatures can be detected via a non-invasive modality such as vaginal fluid screening. Identifying biomarkers of early disease in the fallopian tube may allow for a personalized medicine approach to anti-androgen therapy.

## Data Availability

All IMS data referenced in this article can be found in the MassIVE Database under MSV000082401. LC-MS/MS data on testosterone and extracts can be found on MassIVE under MSV000087039.

## Supplementary Materials

The following are available online at https://www.mdpi.com/article/10.3390/cancers13081925/s1, Figure S1: PTEN deficient cells do not proliferate in response to androgens, Figure S2: MOE:WT and MOE:SCOUTs express equivalent concentrations of total- and phospho-ERK, Figure S3: MEK inhibitor U0126 does not reverse androgen induced invasion through Matrigel, Figure S4: Validation of testosterone presence, Figure S5: Uncropped images of western blots from various figures, Table S1: Antibodies used for immunoblotting, Table S2: Sequences of primers used for qPCR.
