# Supplementary material for: Fallopian Tube-Derived Tumor Cells Induce Testosterone Secretion from the Ovary, Increasing Epithelial Proliferation and Invasion
Source: Cancers (Basel). 2021 Apr 16;13(8):1925. doi: 10.3390/cancers13081925 (PMC8073317; doi:10.3390/cancers13081925)
Supplement: Supplementary file 1 [file cancers-13-01925-s001.zip › cancers-1165186-SI.pdf]

cancers

MDPI

Supplementary Materials

Fallopian Tube-Derived Tumor Cells Induce Testosterone Secretion from the Ovary, Increasing Epithelial Proliferation and Invasion

Jose A. Colina [1], Katherine E. Zink [1], Kanella Eliadis [1], Reza Salehi [2], Emma S. Gargus [3], Sarah R. Wagner [3], Kristine J. Moss [3], Seth Baligod [1], Kailiang Li [1], Brenna J. Kirkpatrick [1], Teresa K. Woodruff [3], Benjamin K. Tsang [2], Laura M. Sanchez [4] and Joanna E. Burdette [1,*]

1 Department of Pharmaceutical Sciences, University of Illinois at Chicago, Chicago, IL 60607, USA; jcolin3@uic.edu (J.A.C.); katherineezink@gmail.com (K.E.Z.); keliad2@uic.edu (K.E.); sbalig2@uic.edu (S.B.); li111@uic.edu (K.L.); brennak@uic.edu (B.J.K.)
2 Department of Cellular & Molecular Medicine and Obstetrics & Gynecology, University of Ottawa, Chronic Disease Program, Ottawa Hospital Research Institute, Ottawa, Ontario, K1Y 4E9, Canada; rsalehi1@ualberta.ca (R.S.); btsang@ohri.ca (B.K.T)
3 Department of Obstetrics and Gynecology, Feinberg School of Medicine, Northwestern University, Chicago, IL 60607, USA; emma.gargus@northwestern.edu (E.S.G.); sarah.wagner@northwestern.edu (S.R.W.); kristine.moss@northwestern.edu (K.J.M.); tkw@msu.edu (T.K.W.)
4 Department of Chemistry and Biochemistry, University of California at Santa Cruz, Santa Cruz, CA 95064, USA; lmsanche@ucsc.edu
* Correspondence: joannab@uic.edu; Tel.: 312-996-6153

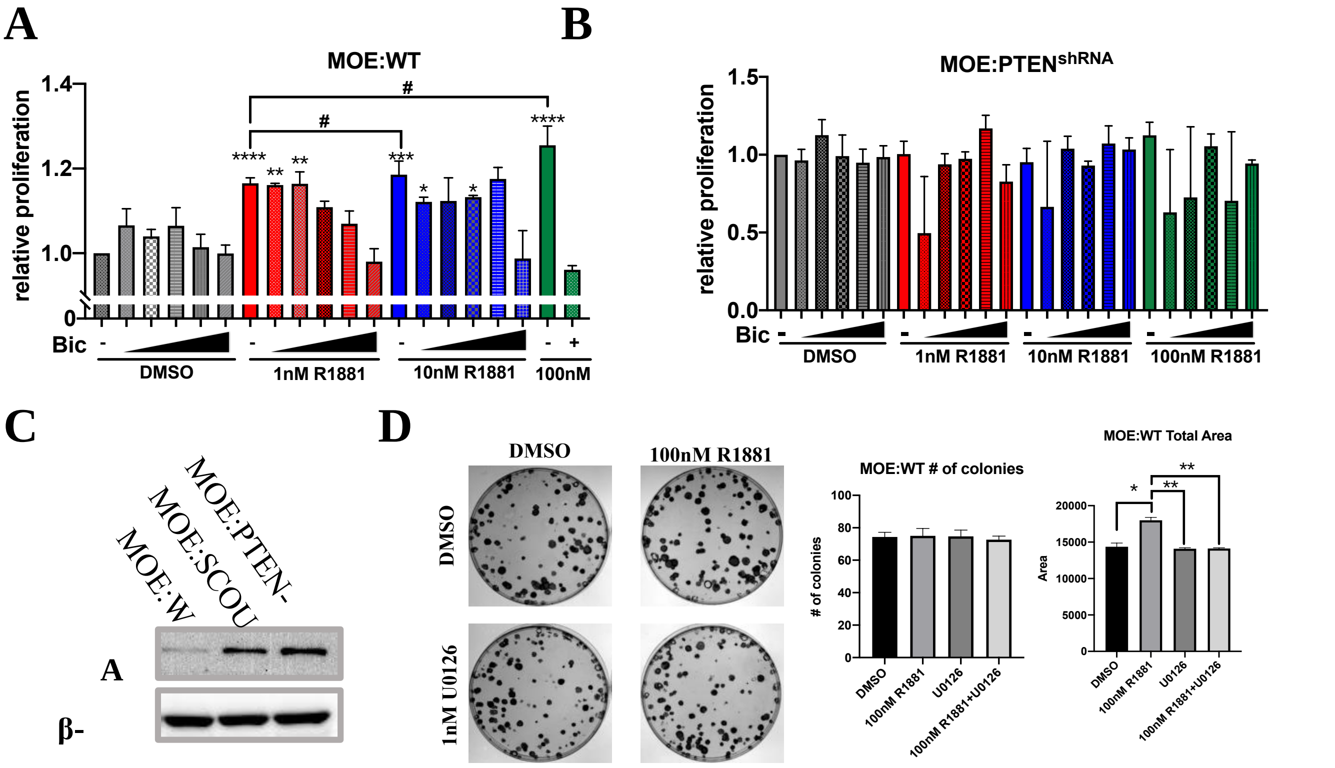

Supplementary Figure S1. PTEN deficient cells do not proliferate in response to androgens. (A,B) Cells were cultured in various concentrations of R1881 and bicalutamide for 5 days. Relative proliferation was measured via Sulforhodamine B (SRB) assay (n≥3, one-way ANOVA followed by a Tukey's post-hoc where p<0.05; additionally, unpaired t-test comparing cells treated with 100nM R1881 to 100nM R1881 + 1µ M U0126 was performed; #P<0.05). Data displayed as mean ± SEM. (C) Representative image of western blot analysis of androgen receptor expression in MOE:WT, MOE:SCOUT, and MOE:PTENshRAN cell lines. (D) representative images and quantification of

colony formation assay (n=3, one-way ANOVA followed by a Tukey's post-hoc where *p<0.05 and **p<0.01).

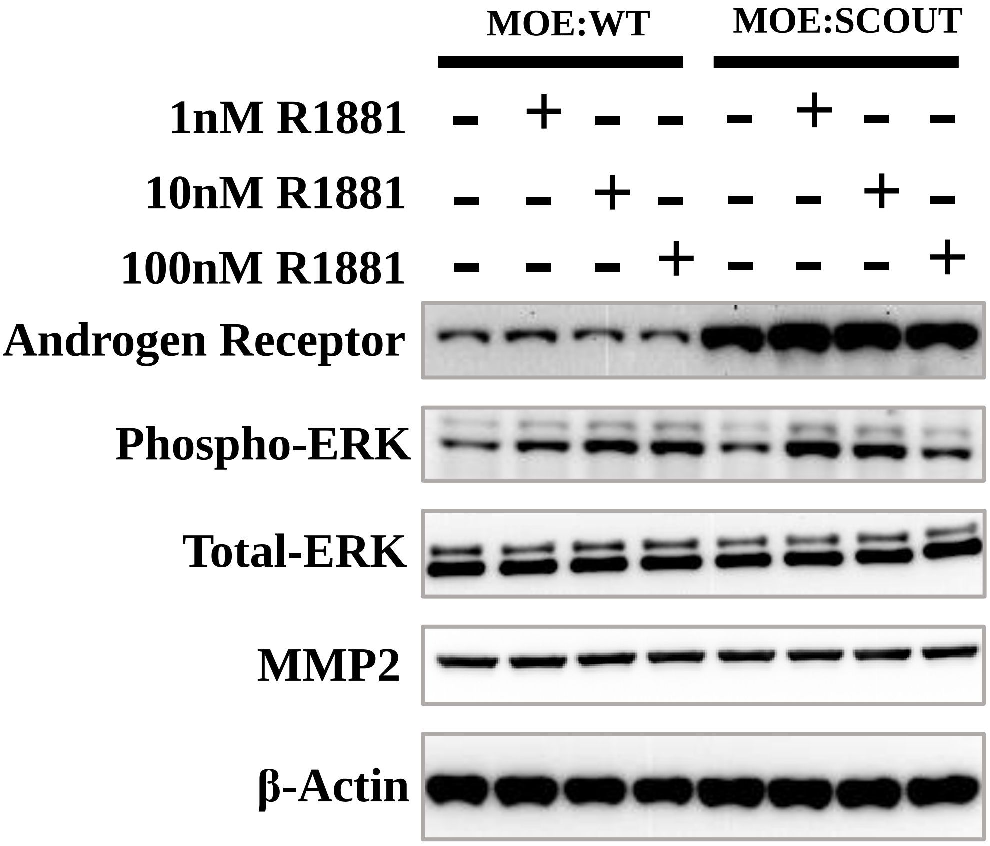

Supplementary Figure S2. MOE:WT and MOE:SCOUTs express equivalent concentrations of total- and Phospho-ERK. Representative image of western blot analysis after cells were treated with various concentrations of R1881.

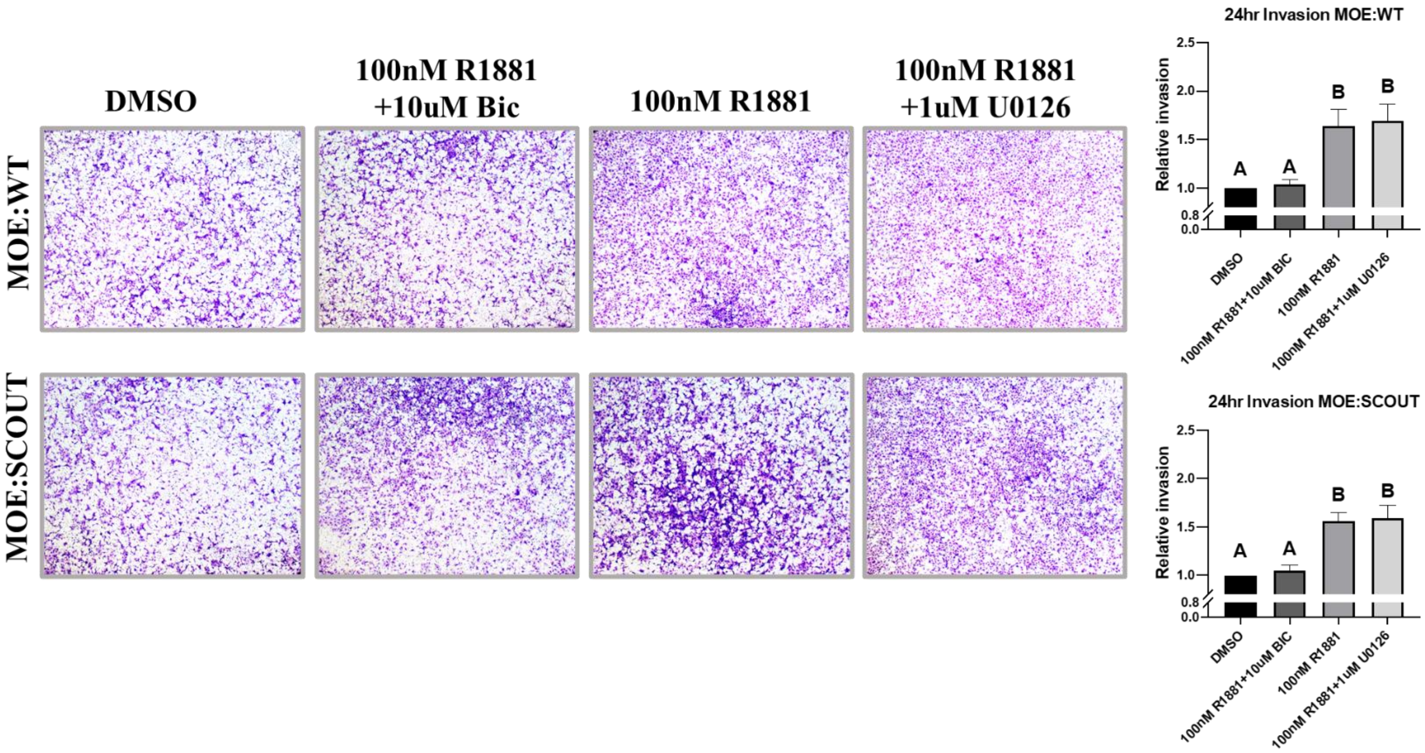

Supplementary Figure S3. MEK inhibitor U0126 does not reverse androgen induced invasion through Matrigel. Cellular invasion was measured by Boyden chamber assay. MOE:WT or MOE:SCOUT cells were seeded in steroid free media on Matrigel coated transwells and allowed to invade toward steroid free media supplemented with either DMSO, 100nM R1881+ Bicalutamide, 100nM R1881, or 100nM R1881+ U0126 for 24hr. (n≥3, one-way ANOVA followed by a Tukey's post-hoc).

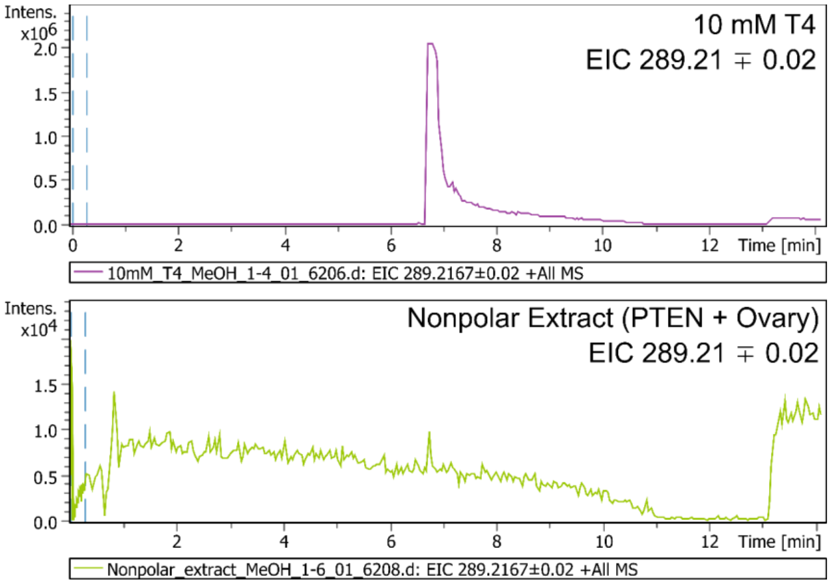

Supplementary Figure S4. The presence of testosterone was validated by an extracted ion chromatogram of the protonated mass (*m*/*z* 289.21 +/- 0.02). In both the testosterone standard (top) and extracted PTEN + ovary sample (bottom), the EIC detected a peak at 6.7 minutes, corresponding to the same mass. The resulting fragmentation can be found in Figure 1.

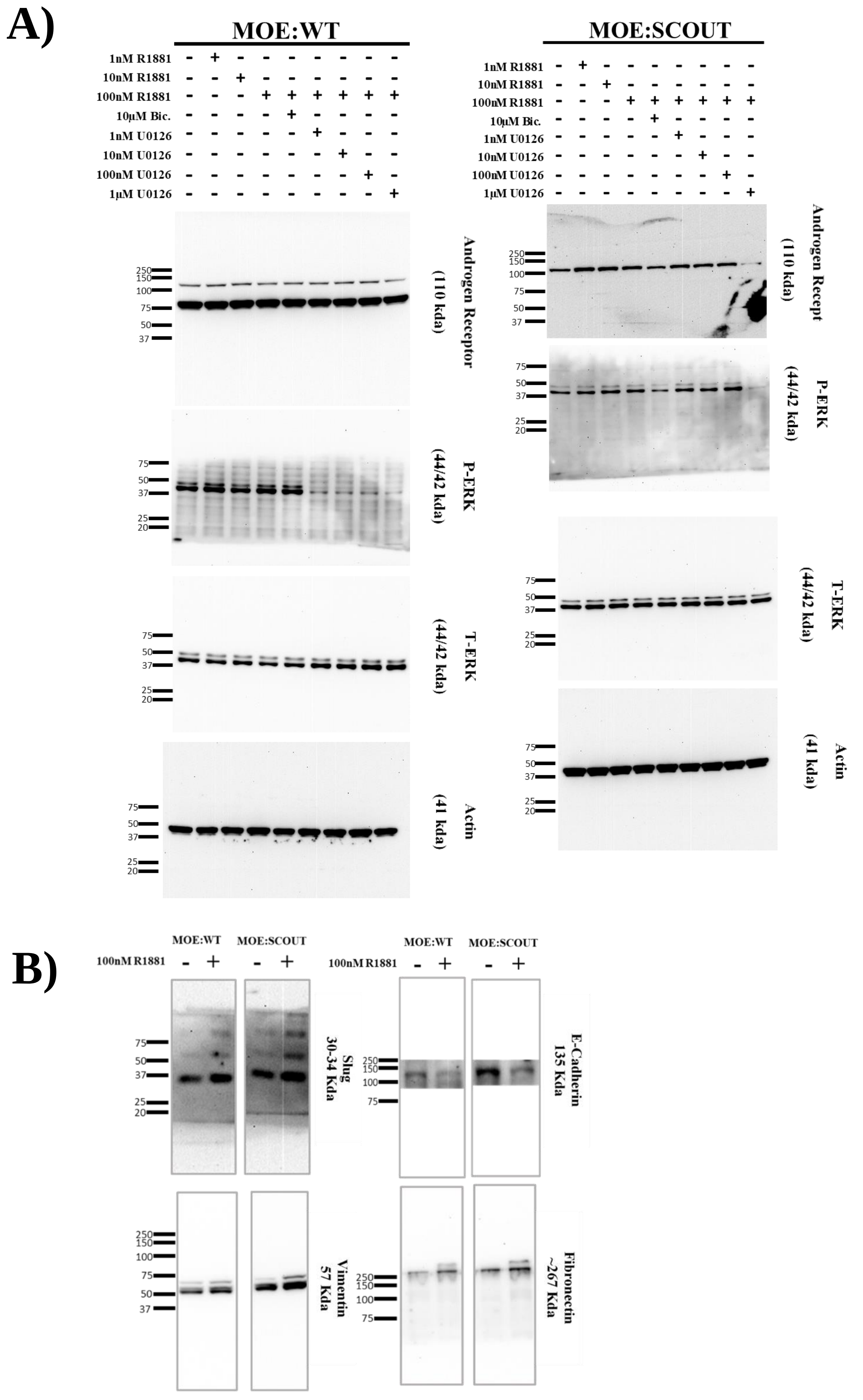

Supplementary Figure S5. Uncropped Western blot images associated with Figure 3 (A) and Figure 4 (B).

Table S1. Antibodies used for immunoblotting.

**Supplemental Table I**		
**Target**	**Cat. Number**	**Manufacturer**
Androgen Receptor	ab133273	Abcam
P-ERK	4370	Cell Signaling
T-ERK	4695	Cell Signaling
SLUG	ab38551	Abcam
Vimentin	5741T	Cell Signaling
E-Cadherin	3195S	Cell Signaling
Fibronectin	F3648	Sigma-Aldrich
Actin	AC026	Abclonal

Table S2. Sequences of primers used for qPCR.

**Supplemental Table II**<br>**Target**	**Primers**
Androgen Receptor	TTATGGGGACATGCGTTTGGA
	GAGAGCTCCGTAGTGACAGC
FKBP5	TGGTGTTCGTTGTTGGGGAA
	CCAAAACCATAGCGTGGTCC
AIG	TTCCCTGTCGGGGTTTTTGT
	ACCACACGTTCAAGCTCTCG
NDRG1	ATGGTAGAGGGTCTCGTGCT
	GGTCTCTCCGGCTGTTGTAG
GREB1	ATGCCCTGCTTGGTTTC
	CTTGAGGTGCTTCTGTTTCT
FOXJ1	GGACTATGCCACCAACCCAC
	TTGTGGCGGATGGAATTCTGC
GAPDH	AGGTCGGTGTGAACGGATTTG
	TGTAGACCATGTAGTTGAGGTCA
